# The stimulatory activity of plasma in patients with advanced non-small cell lung cancer requires TLR-stimulating nucleic acid immunoglobulin complexes and discriminates responsiveness to chemotherapy

**DOI:** 10.1186/s12935-014-0080-1

**Published:** 2014-08-12

**Authors:** Zengguang Xu, Fengying Wu, Chunhong Wang, Xiyu Liu, Baoli Kang, Shan Shan, Xia Gu, Kailing Wang, Tao Ren

**Affiliations:** 1 Department of Scientific Research, East Hospital, Tongji University School of Medicine, Shanghai 200120, China; 2 Department of Preventive Medicine, East Hospital, Tongji University School of Medicine, Shanghai, China; 3 Department of Oncology, Shanghai Pulmonary Hospital, Tongji University School of Medicine, Shanghai, China; 4 Department of Respiratory Medicine, East Hospital, Tongji University School of Medicine, 150 Jimo Road, Pudong New Area, Shanghai 200120, China; 5 Department of Chest Surgery, The Bethune First Hospital of Jilin University, Changchun, China

**Keywords:** Non-small cell lung cancer, Chemotherapy, Toll-like receptor, Immune complex

## Abstract

**Background:**

Therapeutic options for patients with non-small cell lung cancer (NSCLC) are often restricted to systemic chemotherapy. However, the molecular and cellular processes during chemotherapy of advanced NSCLC patients still remain unclear. Here we investigated the stimulatory activity of plasma in advanced NSCLC patients and its correlation with chemotherapy.

**Methods:**

Whole blood samples from advanced NSCLC patients were collected before the first, second, and third cycle of chemotherapy. Plasma was isolated following centrifugation of whole blood. PBMCs were isolated from whole-blood specimens by Ficoll-Hypaque density gradient centrifugation. Immune complexes (ICs) were isolated from NSCLC plasma using the IgG Purification Kit. qRT-PCR was used to detect a broad array of cytokines and chemokines.

**Results:**

The plasma in advanced NSCLC patients was endowed with stimulatory activity and capable of inducing proinflammatory cytokines. Both nucleic acids and immunoglobulin components were required for the stimulatory activity of NSCLC plasma. In consistent, TLR8 and TLR9 conferred the stimulatory activity of plasma in NSCLC patients. Of note, we revealed the decreased stimulatory activity of plasma in patients who responded to chemotherapy.

**Conclusions:**

Our findings demonstrated that the plasma of advanced NSCLC patients required TLR-stimulating nucleic acid immunoglobulin complexes and could discriminate the responsiveness to chemotherapy, which might provide a novel mechanism by which the proinflammatory immune response was induced and a potential new biomarker for evaluating responsiveness to chemotherapy in NSCLC patients.

## Background

Lung cancer is the leading cause of cancer-related death worldwide [1]. Approximately 85% of lung cancers are non-small cell lung cancer (NSCLC), most of which are only diagnosed at advanced stages when therapeutic options are often restricted to systemic chemotherapy [1]–[4]. However, chemotherapeutic treatments for NSCLC are still relatively ineffective [5]. A better understanding of the molecular and cellular processes during chemotherapy of advanced NSCLC patients was urgently needed.

Recent study revealed the presence of endogenous nucleic acid-immunoglobulin complexes in the plasma of cancer patients [6]. It has been known for some time that endougenous nucleic acid-immunoglobulin complexes could induce the proinflammatory responses efficiently [6]–[8]. Accumulating data showed that the plasma proinflammatory cytokines were associated with the progression of NSCLC in patients [5],[9]–[12]. These studies implicated a potential stimulatory effect of plasma in NSCLC patients. However, the stimulatory effect of plasma in advanced NSCLC patients and its correlation with their response to chemotherapy still remain unclear.

Here we reported that nucleic acid-immunoglobulin complexes in plasma from NSCLC patients could effectively induce proinflammatory cytokines through Toll like receptor (TLR) 8 and TLR9. We revealed a reduced stimulatory activity of plasma in patients who responded to chemotherapy. Our findings provided a novel mechanism by which the proinflammatory immune response was initiated and propagated and a promising new method for discriminating the response to chemotherapy in advanced NSCLC patients.

## Results

### The NSCLC plasma could induce proinflammatory response

**Figure 1:**
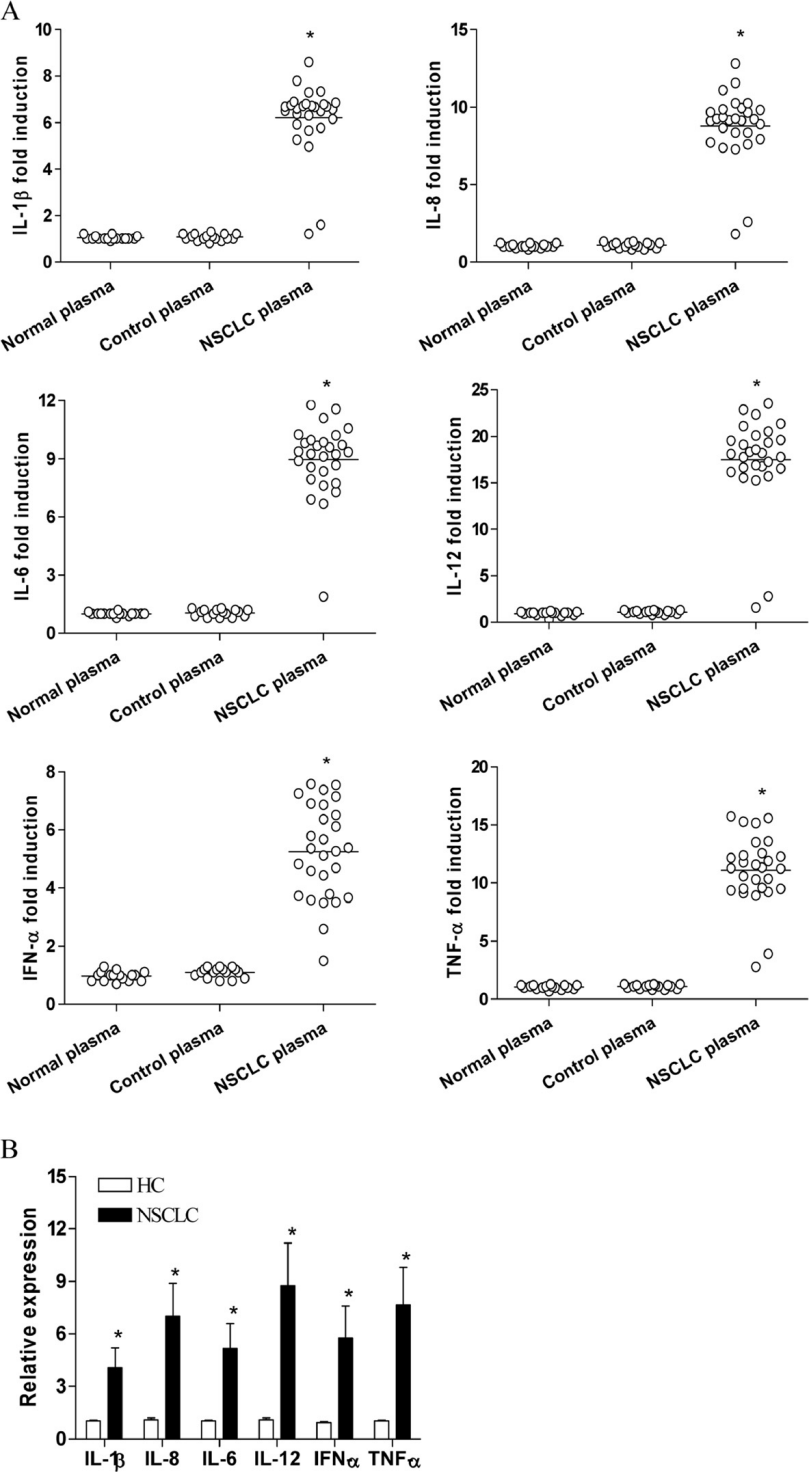
**The plasma of NSCLC patients was endowed with stimulatory activity. (A)** PBMCs derived from normal volunteers were cultured in the presence of 20% plasma derived from normal donors or from NSCLC patients before treatment, or control plasma (20% fetal bovine serum). Data points represented individual patients. *P < 0.001 **(B)** PBMCs derived from 16 normal volunteers or from 28 NSCLC patients were tested for the expression of the indicated cytokines using quantitative PCR. *P < 0.001. Each bar represented the means (±SD) from 16 normal volunteers or from 28 NSCLC patients.

To detect whether the plasma of NSCLC patients was endowed with stimulatory activity, we detected the proinflammatory cytokines generated from PBMCs of healthy controls following exposure to plasma collected from the NSCLC patients prior to chemotherapy. We found that the NSCLC plasma was effectively to induce the production of cytokines including IL-1β, IL-8 IL-6, IL-12, IFN-α and TNF-α (Figure 1A, P < 0.05). In contrast, exposure of normal PBMCs to normal plasma or to control plasma resulted in no significant generation of proinflammatory cytokines (Figure 1A, P > 0.05). Consistently, we analyzed the expression of cytokines in PBMCs isolated from NSCLC patients prior to the treatment, and found that the expression of cytokines in PBMCs was higher in NSCLC patients than that in healthy controls (Figure 1B, P < 0.05).

### Nucleic acid and protein were required for the stimulatory activity of NSCLC plasma

**Figure 2:**
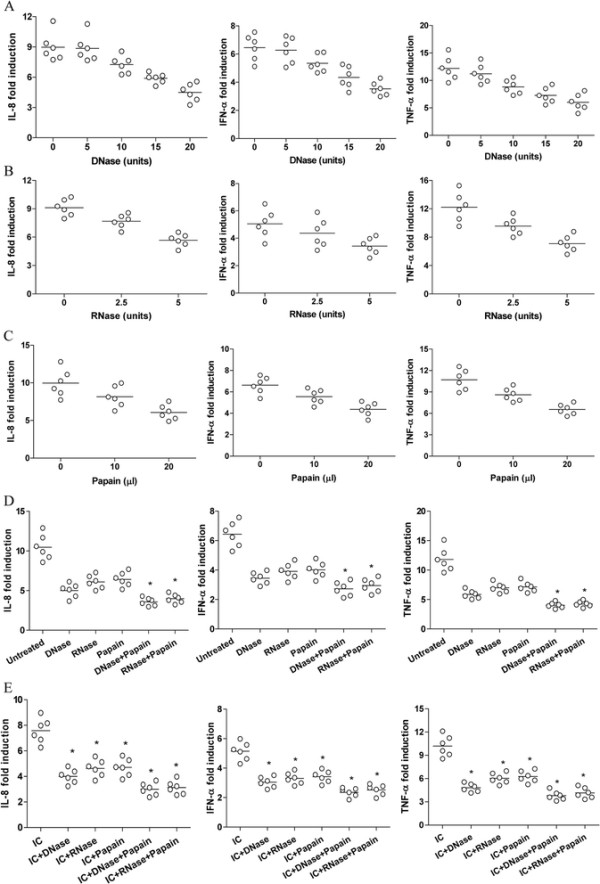
**Nucleic acid and protein in plasma were required for their stimulatory activity. (A-C)** PBMCs derived from normal volunteers were cultured with 20% plasma derived from NSCLC patients before treatment in the presence of the indicated dose of DNase, RNase and Papain respectively. **(D and E)** PBMCs derived from normal volunteers were cultured with 20% plasma derived from NSCLC patients before treatment **(D)** or the isolated immune complexes (IC) **(E)** in the presence of the of DNase (20units), RNase (5units) and Papain (20 μl) respectively. *P < 0.01. Data points represented individual patients.

When plasma from NSCLC patients were pretreated with DNase or RNase and then incubated with the normal PBMCs, as shown in Figure 2A and B, we found that pretreatment of plasma with DNase and RNase could significantly abrogated the induction of IL-8, IFN-α and TNF-α in a dose dependent manner (P < 0.05), suggesting that nucleic acid was crucial for stimulatory activity of NSCLC plasma. Further, pretreatment of plasma with immobilized papain could also inhibit the induction of IL-8, IFN-α and TNF-α in a dose dependent manner (Figure 2C, P < 0.05), suggesting that the immunoglobulin (Ig) component was also important for the stimulatory activity of NSCLC plasma. Besides, when normal PBMCs were incubated with NSCLC plasma pretreated with DNase plus papain or RNase plus papain, the induction of IL-8, IFN-α and TNF-α decreased further (Figure 2D, P < 0.05). These data indicated that nucleic acid-immunoglobulin complexes in plasma might be responsible for the stimulatory effect of NSCLC plasma. Indeed, when normal PBMCs were incubated with ICs isolated from the NSCLC plasma prior to chemotherapy, as shown in Figure 2E, we observed an significant expression of cytokines, which could be abrogated by pretreatment with DNase, RNase and papain (P < 0.05).

### TLR8 and TLR9 conferred the stimulatory activity of NSCLC plasma

**Figure 3:**
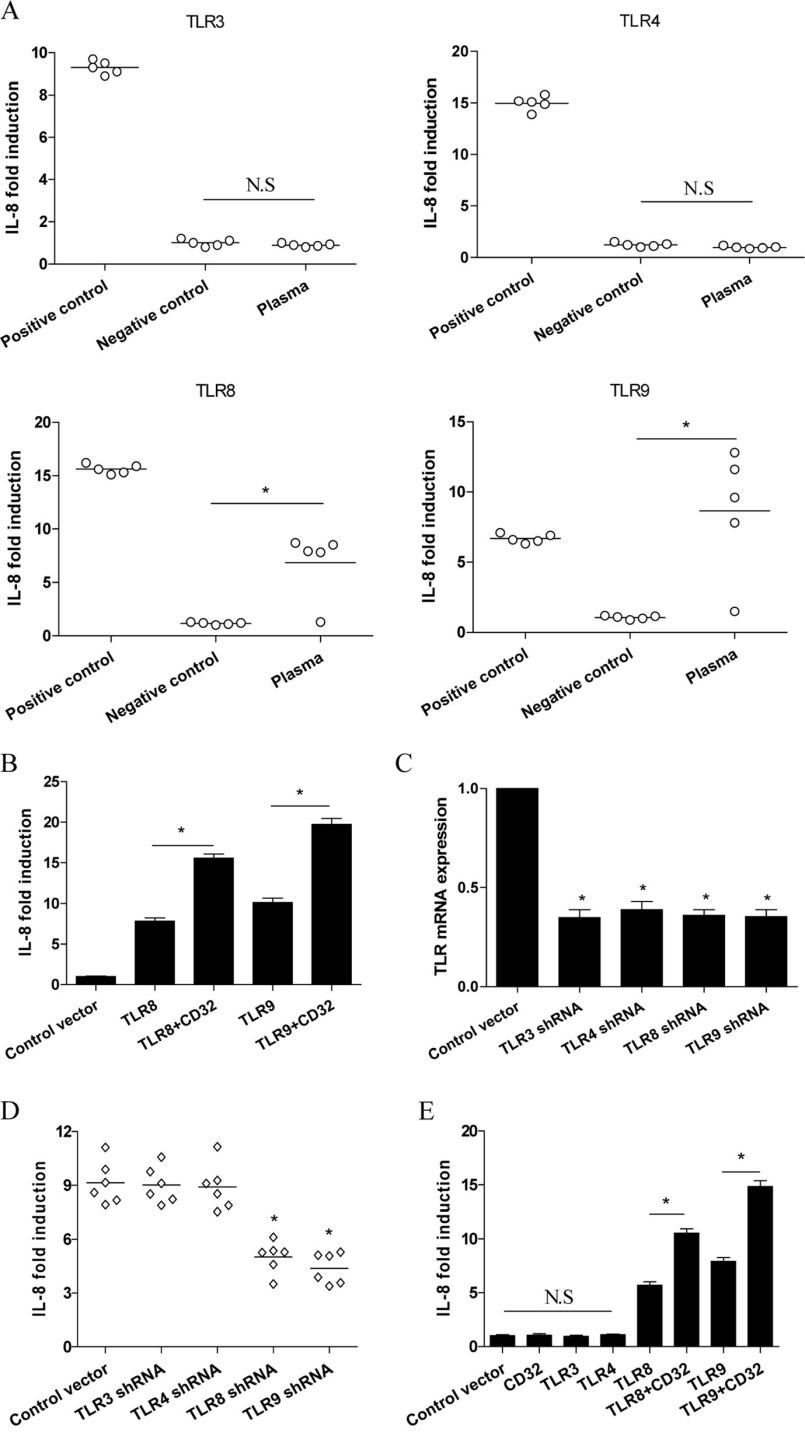
**TLR8 and TLR9 conferred the stimulatory activity of plasma of NSCLC patients. (A)** HEK cells transfected with TLR3, TLR4, TLR8 and TLR9 were incubated with 20% plasma from NSCLC patients before treatment for 6 h, and then assayed for the expression of IL-8 using quantitative PCR. Negative controls were HEK transfectants stimulated with plasma from normal adult volunteers. Positive controls were HEK transfectants stimulated with TLR agonists. **(B)** TLR8 and TLR9-expressing HEK cells were co-transfected with CD32-expression vector and then stimulated with 20% plasma from NSCLC patients before treatment for 6 h. *P < 0.001. **(C and D)** PBMCs derived from normal volunteers were transfected with the indicated TLR shRNA respectively for 24 h, and then cultured with 20% plasma derived from NSCLC patients before treatment for 6 h. The efficacy of TLR shRNA was evaluated by detecting their relative mRNA expression. *P < 0.01. (E) HEK cells transfected with the indicated TLR with or without CD32 were incubated with the isolated IC from NSCLC plasma before treatment for 6 h. *P < 0.001. Data points represented individual patients. Each bar represented the means (±SD) from 5 NSCLC patients.

We then explored the potential role of TLRs in induction of cytokines by NSCLC plasma using human embryonic kidney (HEK) cells transfected with TLR3, TLR4, TLR8, and TLR9 respectively. We found that 4 of 5 NSCLC plasmas effectively induced the expression of IL-8 in HEK cells transfected with TLR8 and TLR9, which could be enhanced by co-transfection with CD32 (Figure 3A and B, P < 0.05). These data suggested that TLR8 and TLR9 were required for the stimulatory effect of NSCLC plasma. To confirm these results, normal PBMCs were transfected with shRNA targeting TLR3, TLR4, TLR8 and TLR9, and then stimulated with the NSCLC plasma collected before chemotherapy. As shown in Figure 3C, we found that transfection with shRNA effectively down-regulated their mRNA expression respectively (P < 0.05). Of important, transfection with shRNA targeting TLR8 and TLR9, but not TLR3 and TLR4, significantly inhibited the induction of IL-8 (Figure 3D, P < 0.05). Finally, we confirmed that ICs isolated from the NSCLC plasma could effectively induce the expression of cytokines through TLR8 and TLR9 but not TLR3 and TLR4 (Figure 3E and data not shown).

### The stimulatory activity of NSCLC plasma was decreased in patients who responded to chemotherapy

**Figure 4:**
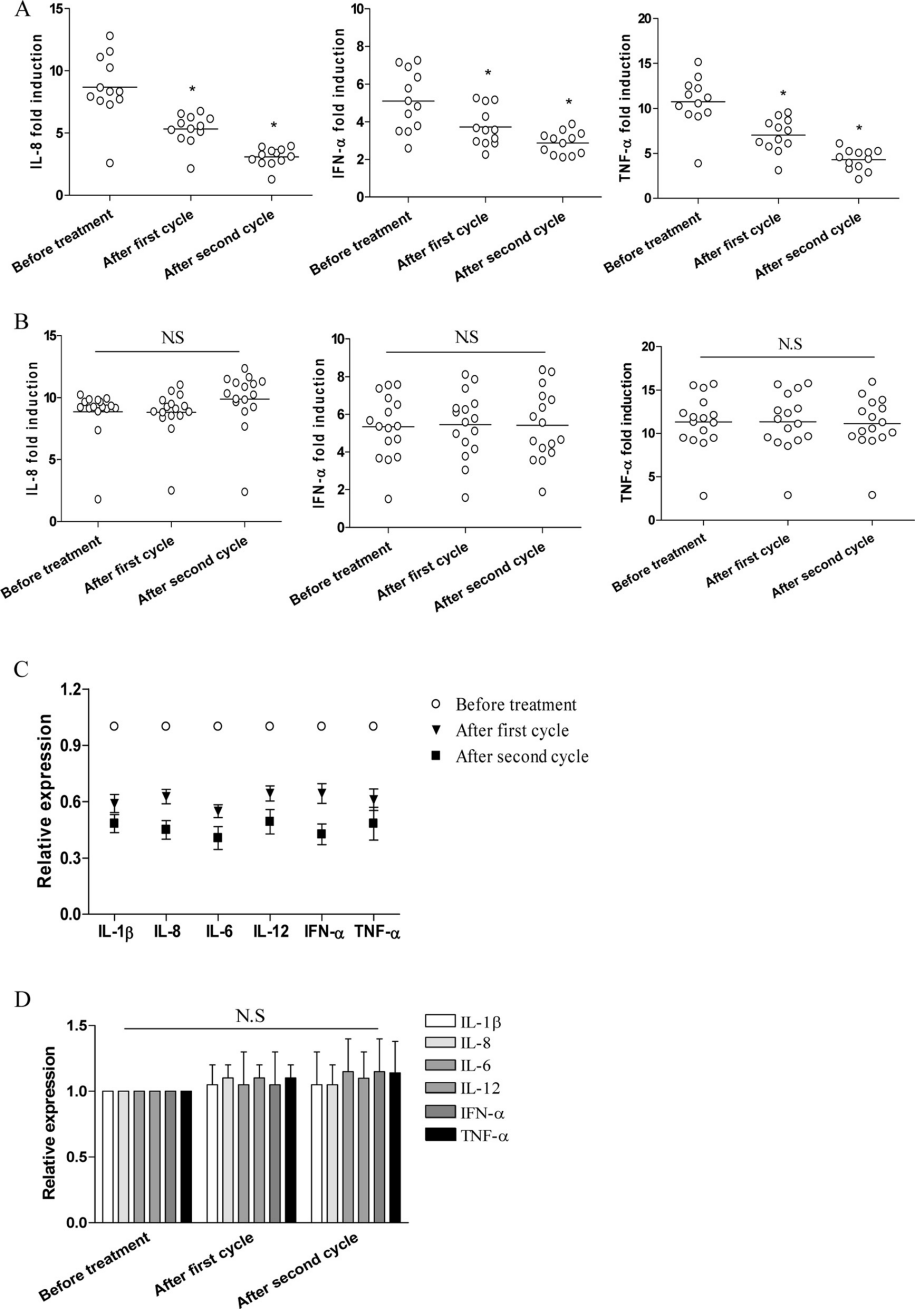
**Reduced stimulatory activity of plasma in patients who responded to chemotherapy. (A** and **B)** PBMCs derived from normal volunteers were cultured with 20% plasma derived from NSCLC patients who responded to chemotherapy **(A)** or not responded to chemotherapy **(B)** before treatment, after the first cycle and after the second cycle of chemotherapy respectively. Data points represented individual patients. *P < 0.01. **(C and D)** PBMCs derived from NSCLC patients who responded to chemotherapy **(C)** or not responded to chemotherapy **(D)** before treatment, after the first cycle and after the second cycle of chemotherapy respectively, and then assayed for their expression of the indicated cytokines. Each bar represented the means (±SD) from 12 responders or 16 nonresponders.

When we analyzed the stimulatory activity of NSCLC plasma in the responders and nonresponders of advanced NSCLC patients to chemotherapy, as shown in Figure 4A, we found that exposure to NSCLC plasma from responsive patients before the second and third cycle of chemotherapy resulted in a decreased induction of cytokines in normal PBMCs. In contrast, plasma isolated from the nonresponders before the first, second and third cycle of chemotherapy induced a generally comparable level of cytokines (Figure 4B, P > 0.05). These findings indicated that the plasma from responders exerted a reduced stimulatory activity. In consistent, we found a decreased expression of cytokines in PBMCs of responders after the first and the second cycle of chemotherapy (Figure 4C, P < 0.05). The expression of cytokines in PBMCs of nonresponders was generally comparable during the first and second cycle of chemotherapy (Figure 4D, P > 0.05).

## Discussion

The discovery of a series of innate immune-specific receptors activated by pathogen associated molecular patterns led to a new understanding of innate immunity mechanisms [13],[14]. Among the innate immune-specific receptors, the best characterized are the Toll like receptors (TLRs), which are primary identified mainly on cells of the immune system, and play an essential role in adaptive immunity [15]–[17]. Recently, accumulating data suggested that TLRs were involved in the development of antitumor immunity [18]–[21]. In lung cancer patients, accumulating evidence suggested that TLR9 signaling played a crucial role in anti-tumor immunity [22]–[24]. However, TLR9 response to CpG oligonucleotide (ODN) in human lung cancer cells could also enhance their growth and invasive potential in vitro and in vivo [25]–[31]. Besides, TLR9 signaling could increase the release of VEGF in a mouse model of lung cancer [32]. Therefore, the potential role of TLRs in tumor immunity was complex and still deserved to be elucidated. Our current study showed that NSCLC plasma could induce the proinflammatory cytokines. Of note, TLR8 and TLR9 signaling contributed to the generation of proinflammatory immune responses in advanced NSCLC patients. In addition, NSCLC plasma effectively induced expressions of pro-inflammatory cytokines from autologous NSCLC PBMCs (Additional file 1: Figure S1). Our findings were consistent with previous studies, which showed that the nucleic acid-immunoglobulin complexes were effect to induce immune response and thus played crucial roles in autoimmune disease and antitumor immunity [6],[7], and provided a novel insight into the production of proinflammatory cytokines in NSCLC patients.

In present study, we revealed a reduced stimulatory activity of plasma in NSCLC patients who responded to chemotherapy. Our data were in line with previous evidence which showed that high levels and insufficient decreases of nucleosomes during the first cycle of chemotherapy indicated poor outcome of NSCLC patients [33], and the previous study which found that the circulating tumor cells and tumor DNA in peripheral blood was associated with clinical response to chemotherapy in NSCLC patients [1]. Our results suggested that reduced chemotherapy resistance was associated with TLR-stimulating nucleic acid immunoglobulin complexes in peripheral blood of advanced NSCLC patients. However, the precise mechanisms by which the nucleic acid-immunoglobulin complexes were generated and involved in antitumor immunity undoubtedly need successive studies.

We should acknowledge some limitations of this study. The clinical sample size in this study was relatively small, and thus, large-scale studies were needed. The precise mechanisms by which the nucleic acid-immunoglobulin complexes were generated and involved in antitumor immunity undoubtedly needed successive studies. In addition, the findings should be tested at individual level to further the translational study.

## Conclusions

Our results suggested that the stimulatory activity of plasma in the form of TLR-stimulating nucleic acid immunoglobulin complexes in patients with advanced NSCLC was associated with their chemotherapy resistance. These findings might provide a novel mechanism by which proinflammatory immune responses were initiated and propagated in NSCLC patients and a potential new method for discriminating the NSCLC patients who responded to chemotherapy.

## Methods

### Ethics statements

This study was approved by the Ethics Committee of Tongji University. The collection and storage of patient medical information was analyzed anonymously. The peripheral blood samples were collected after obtaining written informed consent.

### Patients

A total of 28 patients with newly diagnosed NSCLC (stages III and IV) without infection were recruited, and all the peripheral blood samples were collected after obtaining informed consent. The clinical characteristics of NSCLC patients were summarized in Table 1. Normal controls (n = 16) were recruited and all were in excellent health at the time of the study.

**Table 1:** Characteristics of the advanced NSCLC patients

**Characteristics**	**n=28**
Age (y)	71 ± 76
Gender	
Female	12
Male	16
Stage	
III A	3
IIIB	8
IV	17
Histology	
Adenocarcinoma	11
Squamous carcinoma	9
Not classified carcinoma	8
Mode of therapy	
CMV	14
MV	9
GC	5

*Abbreviations*: CMV caboplatin + mitomycin c + Vinblastin, MV mitomycin c + Vinorelbin, GC gemcitabine + cisplatin.

### Classification of response to chemotherapy

The classification of response to chemotherapy was performed as previously described [33]. Before start of the third cycle of chemotherapy, staging investigations were done consisting of clinical examination, whole body computed tomography, and laboratory examinations. The response to therapy was classified according to the World Health Organization classifications defining “remission” as reduction of the tumor volume ≥50%, “progression” as increase of the tumor volume ≥25% or appearance of new tumor manifestations, and “no change” as reduction of the tumor volume <50% or increase <25%. In this study, 12 patients reached remission (42.9%), 11 suffered from progression (39.3%), and 5 had no change of disease (17.8%). The patients with “progression” and “no change” were joined to the group of nonresponders to therapy, whereas patients with remission were classified as responders to therapy.

### Sample Collection and preparation

Whole-blood samples were collected before the first, second, and third cycle of chemotherapy for determination of their stimulatory effect of plasma. Plasma was isolated following centrifugation of whole blood. PBMCs were isolated from whole-blood specimens by Ficoll-Hypaque density gradient centrifugation (Sigma-Aldrich).

### Detection of cytokines

qRT-PCR was used to detect a broad array of cytokines and chemokines, using gene specific sense and anti-sense primers, as previously described [6],[7]. In experiments using PBMCs, PBMCs (2 × 10^6^/ml) were stimulated with conditioned medium containing 20% of NSCLC plasma (vol/vol). Specifically, 1 million cells in suspension were stimulated with either TLR agonist or 20% patient plasma for 6 hours prior to harvesting cells for RNA extraction. In experiments using TLR-expressing cell lines, cells were seeded at 2 × 10^5^ cells/well, rested in RPMI 1640 overnight, and then exposed to either TLR agonists or 20% patient plasma for the indicated time. Exposure to 20% of a single lot of fetal bovine plasma was used as a negative control. Relative quantification of gene expression was normalized against the housekeeping gene *GAPDH*.

### Isolation of ICs and treatment of plasma with nucleases and protease

Isolation of ICs and treatment of plasma with nucleases and protease were achieved as previously described [6]. Immune complexes (ICs) were isolated from patient plasma using the IgG Purification Kit (Pierce) per the manufacturer’s instructions. To test the role of nucleic acids in the immunostimulatory activity of test plasma, 20 μl patient plasma were treated with 20 units of DNase I (Ambion) or 5 units of RNase (Promega) for 30 minutes at room temperature before stimulation of cells. Protease treatment of test plasma was achieved using 20 μl of immobilized papain (Pierce) for 4 h at room temperature prior to stimulation of cells.

### TLR transfected HEK293 cells and TLR siRNA

HEK293 cells stably expressing human TLR3, TLR4, TLR8, TLR9, and control cells, the TLR agonist as well as the shRNA targeting human TLR3, TLR4, TLR8 and TLR9, were all purchased from Invivogen and used according to the instructions.

### Statistical analyses

Data are presented as the mean ± standard deviation from three independent experiments performed in triplicate. T tests were used for statistical analyses using the program PRISM 6.0 (GraphPad Software Inc., San Diego, CA, USA). P < 0.05 was considered to be significant.

## Competing interests

The authors declare that they have no competing interests.

## Authors’ contributions

Z X and F W carried out experiments and analyzed data. C W participated in study design, performed statistical analysis and helped to draft the manuscript. X L participated in study design and helped to draft the manuscript. B K, S S, X G and K W participated in doing experiments, data analysis and regents supplies. T R conceived of the study, participated in its design and wrote the manuscript. All authors read and approved the final manuscript.

## Additional file

## Supplementary Material

Additional file 1: Figure S1. NSCLC plasma induced expressions of pro-inflammatory cytokines from autologous NSCLC PBMCs. (A) NSCLC plasma was incubated with HC PBMCs or autologous NSCLC PBMCs. respectively and detected for IL-8 and TNF-α expressions. Each bar represented themeans (±SD) from 3 normal controls and 3 NSCLC patients. Click here for file

## References

[B1] Punnoose EA Atwal S Liu W Raja R Fine BM Hughes BG Hicks RJ Hampton GM Amler LC Pirzkall A Lackner MR Evaluation of circulating tumor cells and circulating tumor DNA in non-small cell lung cancer: association with clinical endpoints in a phase II clinical trial of pertuzumab and erlotinib Clin Cancer Res 2012 18 2391 2401 10.1158/1078-0432.CCR-11-3148 22492982

[B2] Holdenrieder S von Pawel J Dankelmann E Duell T Faderl B Markus A Siakavara M Wagner H Feldmann K Hoffmann H Raith H Nagel D Stieber P Nucleosomes and CYFRA 21–1 indicate tumor response after one cycle of chemotherapy in recurrent non-small cell lung cancer Lung Cancer 2009 63 128 135 10.1016/j.lungcan.2008.05.001 18571761

[B3] Jemal A Siegel R Ward E Murray T Xu J Thun MJ Cancer statistics, 2007 CA Cancer J Clin 2007 57 43 66 10.3322/canjclin.57.1.43 17237035

[B4] Mountain CF The international system for staging lung cancer Semin Surg Oncol 2000 18 106 115 10.1002/(SICI)1098-2388(200003)18:2<106::AID-SSU4>3.0.CO;2-P 10657912

[B5] Postel-Vinay S Vanhecke E Olaussen KA Lord CJ Ashworth A Soria JC The potential of exploiting DNA-repair defects for optimizing lung cancer treatment Nat Rev Clin Oncol 2012 9 144 155 10.1038/nrclinonc.2012.3 22330686

[B6] Lin Y Zhang L Cai AX Lee M Zhang W Neuberg D Canning CM Soiffer RJ Alyea EP Ritz J Hacohen N Means TK Wu CJ Effective posttransplant antitumor immunity is associated with TLR-stimulating nucleic acid-immunoglobulin complexes in humans J Clin Invest 2011 121 1574 1584 10.1172/JCI44581 21403403 PMC3069775

[B7] Means TK Latz E Hayashi F Murali MR Golenbock DT Luster AD Human lupus autoantibody-DNA complexes activate DCs through cooperation of CD32 and TLR9 J Clin Invest 2005 115 407 417 10.1172/JCI23025 15668740 PMC544604

[B8] Chiba S Baghdadi M Akiba H Yoshiyama H Kinoshita I Dosaka-Akita H Fujioka Y Ohba Y Gorman JV Colgan JD Hirashima M Uede T Takaoka A Yagita H Jinushi M Tumor-infiltrating DCs suppress nucleic acid-mediated innate immune responses through interactions between the receptor TIM-3 and the alarmin HMGB1 Nat Immunol 2012 13 832 842 10.1038/ni.2376 22842346 PMC3622453

[B9] Enewold L Mechanic LE Bowman ED Zheng YL Yu Z Trivers G Alberg AJ Harris CC Serum concentrations of cytokines and lung cancer survival in African Americans and Caucasians Cancer Epidemiol Biomarkers Prev 2009 18 215 222 10.1158/1055-9965.EPI-08-0705 19124500 PMC2790156

[B10] Katsumata N Eguchi K Fukuda M Yamamoto N Ohe Y Oshita F Tamura T Shinkai T Saijo N Serum levels of cytokines in patients with untreated primary lung cancer Clin Cancer Res 1996 2 553 559 9816203

[B11] Tas F Duranyildiz D Argon A Oğuz H Camlica H Yasasever V Topuz E Serum levels of leptin and proinflammatory cytokines in advanced-stage non-small cell lung cancer Med Oncol 2005 22 353 358 10.1385/MO:22:4:353 16260852

[B12] Songür N Kuru B Kalkan F Ozdilekcan C Cakmak H Hizel N Serum interleukin-6 levels correlate with malnutrition and survival in patients with advanced non-small cell lung cancer Tumori 2004 90 196 200 15237582 10.1177/030089160409000207

[B13] Akira S Uematsu S Takeuchi O Pathogen recognition and innate immunity Cell 2006 124 783 801 10.1016/j.cell.2006.02.015 16497588

[B14] Lee CC Avalos AM Ploegh HL Accessory molecules for Toll-like receptors and their function Nat Rev Immunol 2012 12 168 179 22301850 10.1038/nri3151 PMC3677579

[B15] Kawai T Akira S The role of pattern-recognition receptors in innate immunity: update on Toll-like receptors Nat Immunol 2010 11 373 384 10.1038/ni.1863 20404851

[B16] O'Neill LA Golenbock D Bowie AG The history of Toll-like receptors - redefining innate immunity Nat Rev Immunol 2013 13 453 460 23681101 10.1038/nri3446

[B17] Kawai T Akira S Toll-like receptors and their crosstalk with other innate receptors in infection and immunity Immunity 2011 34 637 650 10.1016/j.immuni.2011.05.006 21616434

[B18] Kutikhin AG Association of polymorphisms in TLR genes and in genes of the Toll-like receptor signaling pathway with cancer risk Hum Immunol 2011 72 1095 1116 10.1016/j.humimm.2011.07.307 21872627

[B19] Hamm S Rath S Michel S Baumgartner R Cancer immunotherapeutic potential of novel small molecule TLR7 and TLR8 agonists J Immunotoxicol 2009 6 257 265 10.3109/15476910903286733 19848448

[B20] Kaczanowska S Joseph AM Davila E TLR agonists: our best frenemy in cancer immunotherapy J Leukoc Biol 2013 93 847 863 10.1189/jlb.1012501 23475577 PMC3656332

[B21] Pinto A Morello S Sorrentino R Lung cancer and Toll-like receptors Cancer Immunol Immunother 2011 60 1211 1220 10.1007/s00262-011-1057-8 21789594 PMC11029286

[B22] Manegold C van Zandwijk N Szczesna A Zatloukal P Au JS Blasinska-Morawiec M Serwatowski P Krzakowski M Jassem J Tan EH Benner RJ Ingrosso A Meech SJ Readett D Thatcher N A phase III randomized study of gemcitabine and cisplatin with or without PF-3512676 (TLR9 agonist) as first-line treatment of advanced non-small-cell lung cancer Ann Oncol 2012 23 72 77 10.1093/annonc/mdr030 21464154

[B23] Yamada K Nakao M Fukuyama C Nokihara H Yamamoto N Sekine I Kunitoh H Ohe Y Ohki E Hashimoto J Tamura T Phase I study of TLR9 agonist PF-3512676 in combination with carboplatin and paclitaxel in patients with advanced non-small-cell lung cancer Cancer Sci 2010 101 188 195 10.1111/j.1349-7006.2009.01361.x 19843072 PMC11158877

[B24] Ren T Wen ZK Liu ZM Qian C Liang YJ Jin ML Cai YY Xu L Targeting toll-like receptor 9 with CpG oligodeoxynucleotides enhances anti-tumor responses of peripheral blood mononuclear cells from human lung cancer patients Cancer Invest 2008 26 448 455 10.1080/07357900701681608 18568766

[B25] Ren T Wen ZK Liu ZM Liang YJ Guo ZL Xu L Functional expression of TLR9 is associated to the metastatic potential of human lung cancer cell: functional active role of TLR9 on tumor metastasis Cancer Biol Ther 2007 6 1704 1709 10.4161/cbt.6.11.4826 17986857

[B26] Xu L Zhou Y Liu Q Luo JM Qing M Tang XY Yao XS Wang CH Wen ZK CXCR4/SDF-1 pathway is crucial for TLR9 agonist enhanced metastasis of human lung cancer cell Biochem Biophys Res Commun 2009 382 571 576 10.1016/j.bbrc.2009.03.072 19302975

[B27] Ren T Xu L Jiao S Wang Y Cai Y Liang Y Zhou Y Zhou H Wen Z TLR9 signaling promotes tumor progression of human lung cancer cell in vivo Pathol Oncol Res 2009 15 623 630 10.1007/s12253-009-9162-0 19319670

[B28] Xu L Wang C Wen Z Yao X Liu Z Li Q Wu Z Xu Z Liang Y Ren T Selective up-regulation of CDK2 is critical for TLR9 signaling stimulated proliferation of human lung cancer cell Immunol Lett 2010 127 93 99 10.1016/j.imlet.2009.10.002 19854217

[B29] Wang C Fei G Liu Z Li Q Xu Z Ren T HMGB1 was a pivotal synergistic effecor for CpG oligonucleotide to enhance the progression of human lung cancer cells Cancer Biol Ther 2012 13 727 736 10.4161/cbt.20555 22617774

[B30] Li Q Li X Guo Z Xu F Xia J Liu Z Ren T MicroRNA-574-5p was pivotal for TLR9 signaling enhanced tumor progression via down-regulating checkpoint suppressor 1 in human lung cancer PLoS One 2012 7 e48278 10.1371/journal.pone.0048278 23133627 PMC3487732

[B31] Xu L Wen Z Zhou Y Liu Z Li Q Fei G Luo J Ren T MicroRNA-7-regulated TLR9 signaling-enhanced growth and metastatic potential of human lung cancer cells by altering the phosphoinositide-3-kinase, regulatory subunit 3/Akt pathway Mol Biol Cell 2013 24 42 55 10.1091/mbc.E12-07-0519 23135998 PMC3530778

[B32] Sorrentino R Morello S Giordano MG Arra C Maiolino P Adcock IM Pinto A CpG-ODN increases the release of VEGF in a mouse model of lung carcinoma Int J Cancer 2011 128 2815 2822 10.1002/ijc.25626 20725994

[B33] Holdenrieder S Stieber P von Pawel J Raith H Nagel D Feldmann K Seidel D Circulating nucleosomes predict the response to chemotherapy in patients with advanced non-small cell lung cancer Clin Cancer Res 2004 10 5981 5987 10.1158/1078-0432.CCR-04-0625 15447981

