# Supplementary material for: The stimulatory activity of plasma in patients with advanced non-small cell lung cancer requires TLR-stimulating nucleic acid immunoglobulin complexes and discriminates responsiveness to chemotherapy
Source: Cancer Cell Int. 2014 Aug 12;14:80. doi: 10.1186/s12935-014-0080-1 (PMC4364047; doi:10.1186/s12935-014-0080-1)
Supplement: Additional file 1: Figure S1. — NSCLC plasma induced expressions of pro-inflammatory cytokines from autologous NSCLC PBMCs. (A) NSCLC plasma was incubated with HC PBMCs or autologous NSCLC PBMCs. respectively and detected for IL-8 and TNF-α expressions. Each bar represented themeans (±SD) from 3 normal controls and 3 NSCLC patients. [file s12935-014-0080-1-S1.pdf]

Supplementary Figure 1

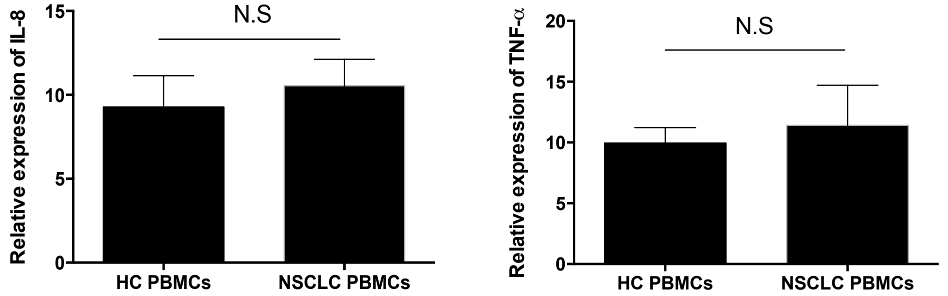

Figure S1 NSCLC plasma induced expressions of pro-inflammatory cytokines from autologous NSCLC PBMCs.

(A) NSCLC plasma was incubated with HC PBMCs or autologous NSCLC PBMCs respectively and detected for IL-8 and TNF-α expressions. Each bar represented the means (±SD) from 3 normal controls and 3 NSCLC patients.
